# Natural history of social and sexual behavior in fruit flies

**DOI:** 10.1038/s41598-020-79075-7

**Published:** 2020-12-14

**Authors:** Reuven Dukas

**Affiliations:** Animal Behaviour Group, Department of Psychology, Neuroscience and Behaviour, McMaster University, 1280 Main Street West, Hamilton, ON L8S 4K1 Canada

**Keywords:** Animal behaviour, Behavioural ecology

## Abstract

The past 2 decades have seen fruit flies being widely adopted for research on social behavior and aggression. This fruitful research, however, has not been well tied to fruit flies’ natural history. To address this knowledge gap, I conducted a field study. My goal was to inform future research conducted in artificial surroundings, and to inspire new investigations that can rely more heavily on fruit flies’ actual natural behavior. My two main novel findings were first, that flies in the field showed significant sociability, as they formed social groups rather than dispersed randomly among fruits of similar quality. Second, males showed fair levels of aggression towards each other as indicated by a lunging rate of 17 per hour, and lower rates of wing threat and boxing. Courtship was the most prominent activity on fruits, with females rejecting almost all males’ advances. This resulted in an estimated mating rate of 0.6 per female per day. Flies showed a striking peak of activity early in the mornings, even at cold temperatures, followed by inactivity for much of the day and night. Flies, however, handled well high temperatures approaching 40 °C by hiding away from fruit and concentrating activity in the cooler, early mornings. My field work highlights a few promising lines of future research informed by fruit flies’ natural history. Most importantly, we do not understand the intriguing dynamics that generate significant sociability despite frequent aggressive interactions on fruits. Males’ responses to female rejection signals varied widely, perhaps because the signals differed in information content perceived by flies but not humans. Finally, flies tolerated cold early mornings perhaps owing to fitness benefits associated with increased mating and feeding opportunities at this time. Flies were adept at handling very high temperatures under the natural daily temperature fluctuations and availability of shelters, and this can inform more realistic research on the effects of global warming on animals in their natural settings.

## Introduction

Fruit flies (*Drosophila melanogaster*) have served as a leading model system in many branches of biology^1–3^, and the twenty-first century has seen them being successfully adopted for research on social behavior as well^4–7^. Given the fundamental similarity among animals, research on social behavior in fruit flies helps us understand social behavior in all animals including humans. Furthermore, such research provides us with essential insights on the mechanisms that underlie social disorders^8–11^.

Research on social behavior may be categorized into aggression, defined as the use of physical force against conspecifics, and sociability, characterized as the tendency to engage in friendly activities with conspecifics. Such activities may include feeding together, traveling in a group, and communal resting or sleeping. While there had been early mentions of fruit fly aggression^12–14^, research on the topic has intensified relatively recently owing to their adoption for neurobiological and genetic analyses of aggression^5,6,15^. Similar to aggression, hints of fruit fly sociability existed for a long time, but focus on the topic is recent. The characterization of cis vaccenyl acetate (cVA) as an aggregation pheromone of fruit flies^16^ implied social attraction, which led to research on its adaptive significance^17^ as well as its mechanisms of action^18,19^. Recent research on sociability in fruit flies has indicated social synchronization of the circadian clock^4^, reliance on social information gleaned from conspecifics^20,21^, the formation of social groups^22–26^ and collective response to threat^27,28^.

To fully understand the evolutionary biology of social behavior, we must link it to its fitness consequences through its effects on access to food, shelter and mates. Laboratory-based analyses of fruit fly social behavior indeed include some of these factors in their protocols. While such protocols seem reasonable, they are, unfortunately, not based on established knowledge about fruit fly social behavior in natural settings. The fundamental reason for this discord is the striking rarity of field work quantifying the natural history of fruit flies^29^. Very few researchers have systematically documented fruit fly behavior in the field, and the majority of those who did focused on sexual behavior. In his review of courtship behavior in *Drosophila*, Spieth^30^ devoted two pages to describing behavior in the field, which were probably based on his own unpublished observations. Markow and colleagues conducted perhaps the most elaborate field work on *D. melanogaster* as well as a few other fruit fly species^31–33^. Other useful field studies include Partridge et al.^34^ and Soto-Yéber et al.^35^. Remarkably, however, 33 years after Partridge et al.^34^ noted that they did not observe fly aggression in their study and hence “the role of fighting in the field remains mysterious”, and in spite of the upsurge in research on fruit fly aggression, the natural history of combat in fruit flies is still an enigma. Similarly, we know nothing about fruit flies’ sociability in nature.

To help alleviate the striking lack of information about the natural history of social behavior in fruit flies, I conducted a field study that included live observations as well as 60 h of video recording to address the following questions. First, what is the social organization of flies at fruit and away from fruit? Specifically, do flies prefer to feed and rest together? Second, how frequent is aggression between males, between females, and between males and females? Do males rely on aggression to monopolize fruit? Are males aggressive towards other males while pursuing females? How do receivers of aggression respond? While my focus was sociability and aggression, I also wished to gather information about other important fruit fly natural history features that we know too little about. Specifically, I asked what is the daily pattern of fly activity and distribution? What is the frequency of males pursuing females? How do females respond to males? What is the frequency of mating? Finally, how often do females and males approach copulating flies to potentially glean information relevant to mate choice copying?

## Results

### General

**Figure 1:**
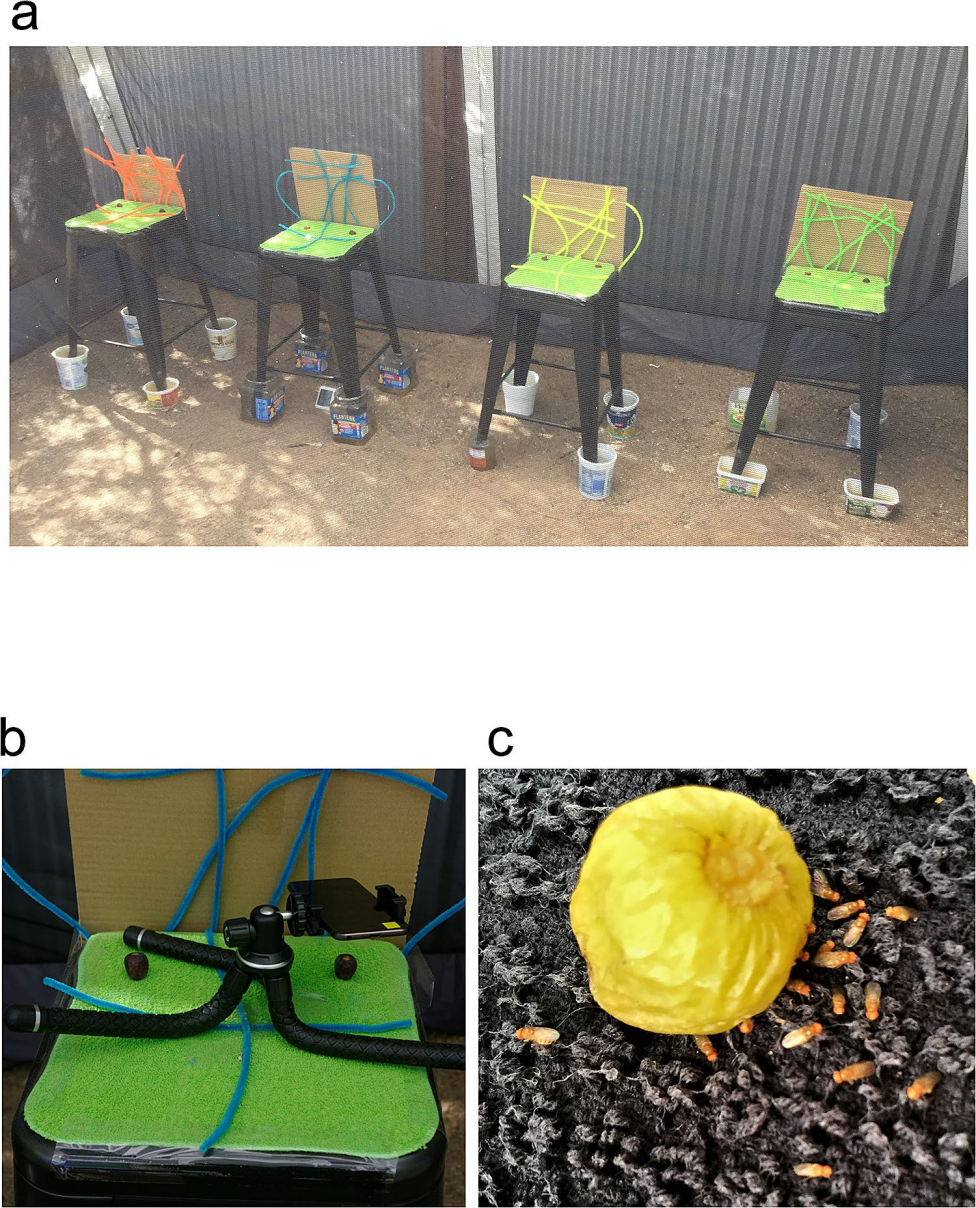
(**a**) The screen enclosure used in the observations was 3 m wide, 3.6 m long and 2.15 m high, and contained 4 stools, each with 2 grapes at the top. The ~ 50 flies inside the enclosure were free to choose their preferred activities and locations. (**b**) A close-up of a stool with 2 grapes and an iPod used for video recording. (**c**) A resting group of 18 flies near a grape at noon, when the temperature was 33 °C. This group constituted about 40% of the flies in this setting and each fly could choose to rest anywhere in the enclosure as well as at 7 other grapes.

**Figure 2:**
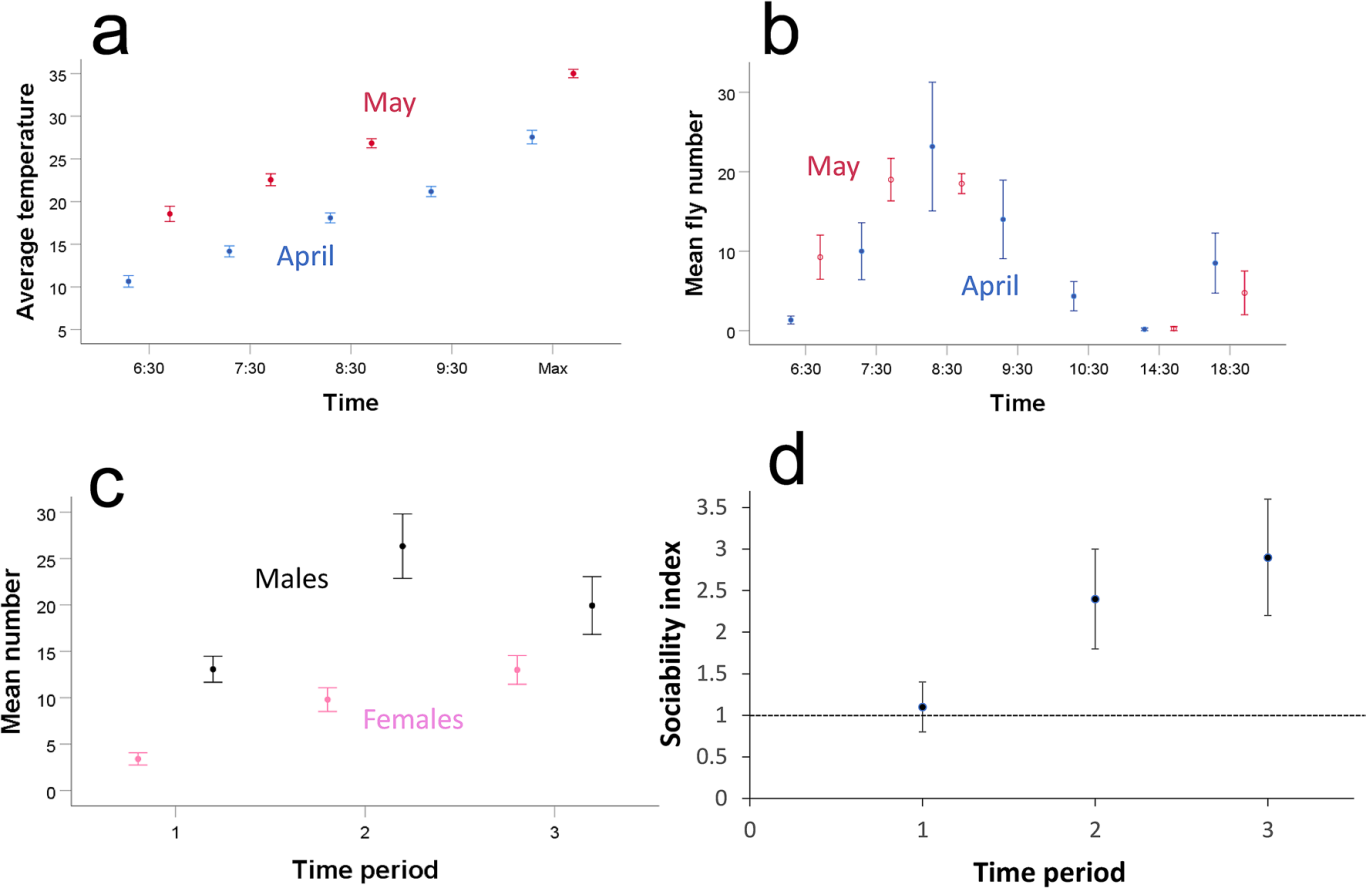
(**a**) The average (± SE) temperature during the mornings of observations and the average (± SE) maximum temperature (marked as “Max” on the X legend) for the same days (n = 6 days in April and 9 days in May; note that I did not record the 9:30 AM temperature in May). (**b**) The average (± SE) number of flies at the fruits during the whole-day scans (n = 5 days; note that I did not record fly numbers at 9:30 AM and 10:30 AM in May). (**c**) The average (± SE) number of flies at the fruits during the 3 morning periods (n = 15 days). These morning periods were at 7:30, 8:30 and 9:30 in the two April replicates and at 6:30, 7:30 and 8:30 in the three May replicates. (**d**) The average (± 95% CI) sociability index during the 3 morning periods. A value of 1 shows random fly distribution while a value significantly above 1 indicates fly sociability. Data are back-transformed estimated marginal means from the GLMM (n = 15 days).

There were strong daily dynamics of fly presence and activity at the fruits. Flies spent much of the day in hiding, mostly away from fruits but sometimes just at the base of fruits (Fig. 1c). I occasionally observed resting groups of various sizes (Fig. 1c), but most often, flies were out of sight. At dawn, fly numbers at fruit was close to zero. Males were typically the first to arrive at fruits. Fly activity peaked very early in the morning regardless of the relatively low temperatures (Fig. 2). For example, during the first replicate in mid-April, I observed vigorous fly activity at 7:30 AM, when the temperatures over the 3 days ranged between 12 and 13.5 °C. Fly activity declined significantly already by early morning, 9:30 AM in April and 8:30 AM in May.

Notable activities at fruits were patrolling, courtship and aggression by males, and feeding, egg laying and evading males by females. Patrolling consisted of males walking or running on the fruit, quickly inspecting any fly, often with brief courtship displays directed at both males and females. Overall, flies spent much of the time cohabiting fruit with no overt interactions. Males spent 0.14 ± 0.02 of the time courtship females and 0.008 ± 0.001 of the time in brief aggressive interactions, while females almost never interacted (see below). The reduced fly activity by early morning was followed by a gradual departure of flies from fruits, which typically culminated with no flies at fruits late in the morning and in the afternoon (Fig. 2b). While flies rested during the peak heat of days in May, in which temperatures approached 40 °C, they resumed activity on the subsequent mornings with no apparent detriment or mortality. I observed a slight increase in fly numbers at fruit but little activity other than feeding at dusk (Fig. 2b).

The analysis of the 3 early morning periods from all 15 days of the 5 replicates indicated that fly numbers at the grapes increased during the morning. There were more males than females on grapes but no change in fly numbers over the 3 days (GLMM, *F*_2,8_ = 6.6, *P* < 0.05 for the morning period, *F*_1,4_ = 15.6, *P* < 0.02 for sex, *F*_2,8_ = 2.2, *P* = 0.18 for day; Fig. 2c).

### Sociability

Flies showed significant sociability during the second and third morning scans (Fig. 2d; Supplementary Video S1 0:06). The increase in fly number from the first to later scans (Fig. 2c) was associated with a significant increase in sociability scores (GLMM, F_2,16_ = 14.3, *P* < 0.001 for the contrast between the first and later periods; Fig. 2d). The turnover rate on grapes was rather high: males and females arrived at the rate of 11.2 ± 3.3 and 8.1 ± 1.3 per grape per h respectively, and departed at the rate of 9.9 ± 3.1 and 6.4 ± 1.4 per grape per h respectively.

### Male–male aggression

**Figure 3:**
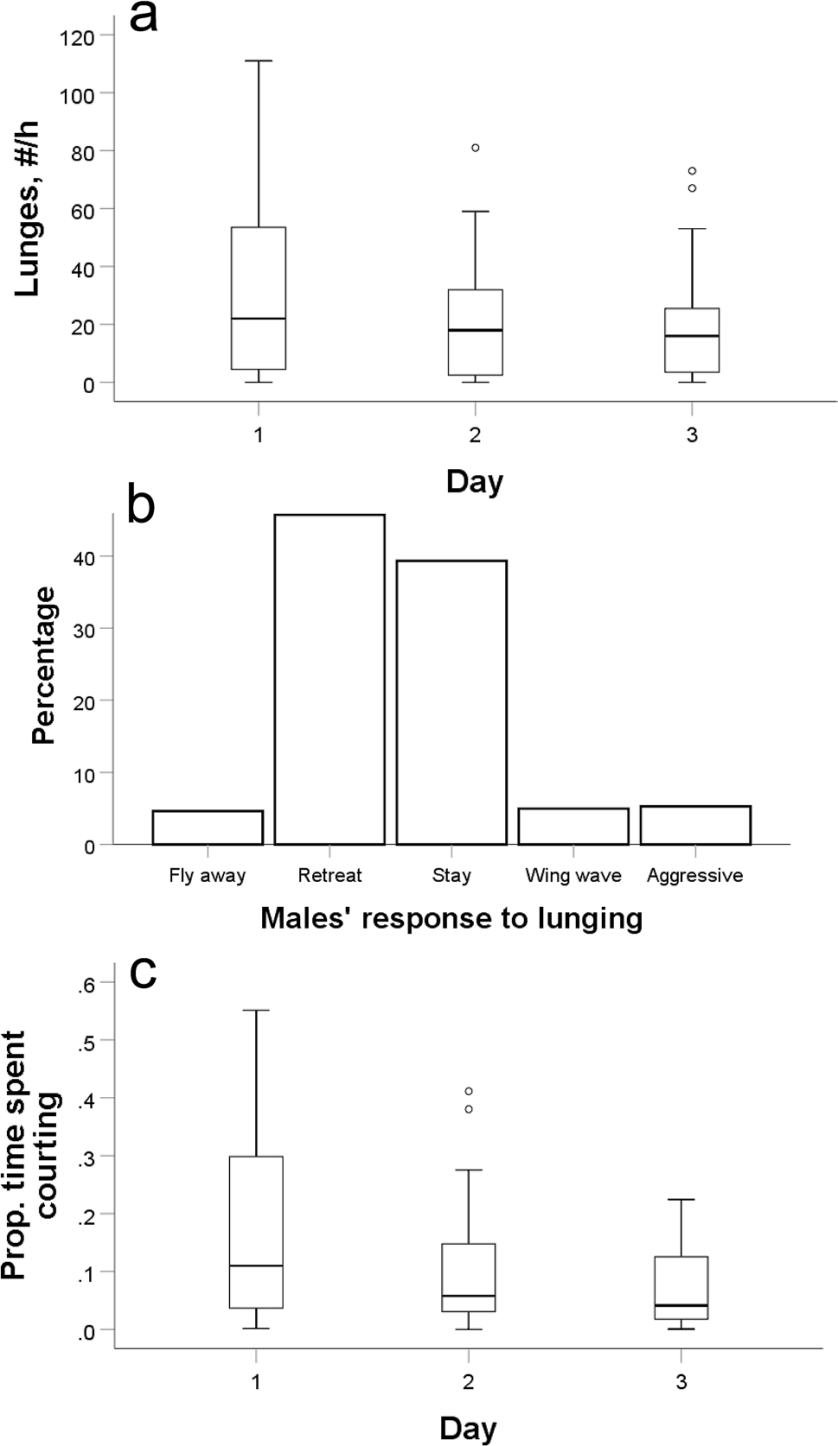
(**a**) Male–male aggression over 3 days depicted as the number of lunges per h at each fruit. The horizontal lines in the boxplots show the medians, the boxes contain the middle 50% of data (interquartile range, IQR), the whiskers above and below each box represent values within ± 1.5 of the IQR, and points depict outliers (n = 59 h of video recordings). (**b**) The types of males’ responses to an attack by another male (n = 905 cases). (**c**) The proportion of time males spent courting females over 3 days (n = 59 h of video recordings).

There was a substantial amount of male–male aggression on fruits, and the median number of lunges was 17 per h. Aggression varied widely, with 17% of the grapes having no lunges and 15% having more than 50 lunges per hour. Aggression declined slightly and not significantly over the 3 days but varied significantly among the 5 replicates (GLMM, F_2,8_ = 2.8, *P* = 0.12 for day, F_4,8_ = 14.8, *P* < 0.001 for replicate; Fig. 3a; Supplementary Video S1 0:51). There were occasional cases of aggression escalating into boxing matches. The majority of grapes (73%) had no boxing, 17% had 2 boxing events per h, and 3.5% had more than 5 boxings per hour.

I examined how males subjected to lunging responded to the aggressors (Fig. 3b; Supplementary Video S1 0:51). The males flew away in less than 5% of the cases but retreated while staying on the fruit in about 45% of the events. In other 45% of the cases, the males either did not show an obvious response or performed wing-waving. Finally, about 5% of lunging events escalated into an aggressive act by the receiver (wing threat, lunging or boxing; Fig. 3b).

In addition to the male–male aggression reported above, I recorded 2 types of male–male aggression when one of the males was engaged in courtship. First, I saw 5 cases in which males took over courtship from another male (Supplementary Video S1 2:52). Second, I observed 7 cases where males lunged at courting males, which led to the males aborting courtship in about half of these cases. Finally, I also observed two incidents of males lunging at copulating males.

To assess a possible link between male aggression and fitness, I examined fly interactions prior to matings (see below). In 50% of the matings, the successful males showed prior aggression. The aggressive males either excluded other males from the top of the fruit (40%), or interfered with and took over another male’s courtship (10%; Supplementary Video S1 2:52).

### Aggression between females and between males and females

I observed only 4 instances of female-female aggression during 4 distinct replicates (Supplementary Video S1 3:29). Males lunged at females a total of 17 times during 4 replicates.

### Sexual behavior

I recorded an average of 29 ± 3.2 male–female courtship bouts per hour. Males spent 0.14 ± 0.02 of the time courting females and showed a non-significant decrease in courtship over the 3 days (GLMM, F_2,8_ = 1.6, *P* = 0.26 for day, F_4,8_ = 0.38, *P* = 0.8 for replicate; Fig. 3c; Supplementary Video S1 4:00). Females had a rich repertoire of responses to pursuing males though they often merely appeared to ignore them. Out of the recorded responses, they extruded their ovipositors in 68% of the courtship bouts, waved their wings in 9%, and flew away to avoid males in 22% of the bouts. During feeding, females often pre-empted approaching males and extruded their ovipositors prior to any courtship. This was typically sufficient to avert further male pursuit. Females’ primary behavior at fruits was feeding, egg laying and rejecting males. Male–male courtship was prevalent (Supplementary Video S1 5:05). My comparison of the rate of male–male courtship in 1 h revealed 19 male–male courtship bouts compared to 69 male–female courtship bouts, a 0.3:1 ratio.

The mating rate was only 0.19 matings per h (11 matings in 59 h of video) and the average mating duration was 25 ± 1.6 min. My live scans indicated that all but 1 mating occurred at fruits, with peak matings (60%) at 7:30 AM (n = 15 matings). In 20% of the cases, newly arrived females initiated mating within a few s with the first males that encountered them. In 70% of the cases, females initially rejected the pursuing males by raising their abdomens or extruding their ovipositors.

To assess the potential for mate-choice copying in natural settings, I scored all approaches to copulating flies. Females very rarely approached mating pairs. They did so only in 30% of the matings while incidentally coming close to the mating couples during feeding (approach rate: 0.024 ± 0.014 per min, mean duration: 12.2 ± 2.2 s). In contrast, males contacted copulating flies at the rate of 1.1 ± 0.4 per min. The males’ contacts were always in the context of their search for mates, and they typically briefly courted the mating females at an average duration of 3.3 ± 0.3 s.

## Discussion

### Sociability and aggression

My two most important findings were, first, that fruit flies under natural settings displayed significant sociability, as indicated by the flies forming social groups while feeding and seeking mates at fruits (Fig. 2d). Second, males showed fair levels of aggression (Fig. 3a). It is important to note that, unlike almost all aggression studies in fruit flies, which isolate individuals from eclosion to the aggression test in order to increase fly aggression rate^5,36^, my natural history work involved flies naturally interacting with each other for a few days post eclosion. That is, there was no artificial enhancement of fly aggression levels. Although I recorded only a small number of matings, I could directly link half of them to males that had been previously aggressive towards other males. The dynamics of social behavior in the field and the tension between sociability and aggression are intriguing and will require further studies. Meanwhile, my observations suggest rapidly changing social settings, with flies assessing different fruits and choosing to stay or leave at any moment based on their interactions with other flies. Most notably, the average hourly arrival and departure rates of flies per fruit, which ranged from about 11 to 6 respectively (see “Results”), represented a large proportion of the total flies observed at all fruits (Fig. 2c).

Some males clearly left fruits owing to aggression from other males. The majority of males (~ 95%), however, remained after being subjected to aggression (Fig. 3b). It thus appears that most aggressive males either could not evict other males from fruits, or chose not to attack them further. Unlike animals with weapons, male fruit flies cannot injure other males. While there is some cost to receiving aggression^37^, it is likely costly to the aggressor as well. Hence perhaps males limit their aggression only for establishing their dominance so they can focus on pursuing females. The fact that most males subjected to aggression stayed on the fruit also suggests that they often preferred to be subordinate on the current fruit than to seek alternatives, perhaps because the current fruit offered a small yet higher likelihood of mating than alternatives. Indeed, in 20% of the matings, I observed females commencing copulation with the first males that approached them within a few seconds after alighting on the fruit. In these cases, the males were neither the sole males on the fruit nor the dominant ones, and it appeared that their mating success had a strong element of chance. Aggressive males, however, were the successful ones in half the matings I observed. This is consistent with our controlled laboratory experiments^38^. Similar to the males, some females (~ 20%) left the fruit after experiencing aggressive pursuit from one or more males. The vast majority of females (~ 80%), however, deflected well pursuing males, primarily by ovipositor extrusion.

Overall then, it seems that the significant, low levels of sociability scores I recorded in the field (Fig. 2d) reflected specific fruit settings where females and males were more likely to stay than to leave owing to lower levels of both males’ aggressive pursuit of females and male–male aggression. It is also possible that a slightly larger group of flies at a fruit led to higher arrival rates of flies owing to social attraction^26,39,40^. In uncontrolled natural environments, we would expect to see aggregations of flies only at the fruits that provide the preferred abiotic and biotic conditions. To avoid apparent sociability driven by pure preference to a subset of fruits, I provided controlled setting with 8 very similar fruits placed on identical substrate. It is possible, however, that flies and microbes generated some variation in fruit quality over time.

Female–female aggression was rare and subtle compared to male–male aggression. It was most likely associated with the aggressor protecting or attempting to gain access to an attractive spot on the fruit. I also recorded the novel behavior of males lunging at females. Some of these lunges may have been owing to males confusing females with males as they were temporally associated with male–male aggression. In other cases, however such lunges were associated with the males courting the females. The function of these lunges, if any, is unclear.

### Sexual behavior

By far the most prevalent activity on fruits was males’ incessant pursuit of females (Fig. 3c), which almost always rebuffed them (Supplementary Video S1 4:00). On average, a female was pursued by a male once every 2 min. Often it was the same male approaching the female over and over in spite of the female’s obvious rejection signals. Females most frequently seemed to ignore pursuing males though it is possible that I did not perceive subtle or invisible responses. The most efficient way that females could avoid males was flying away. This had the obvious cost of leaving an attractive feeding and egg laying site. While females could land on another fruit, it is not clear whether it could improve their ability to evade males. Hiding away from fruit most likely consisted a secure setting for females and probably explained the lower number of females than of males at fruits (Fig. 2c).

Females’ ovipositor extrusion varied in its effect on males. Sometimes it seemed to have a strong repellency effect while other times, males kept the pursuit. Although well documented^41–43^, the signals conveyed by ovipositor extrusion are unknown. Intriguingly, two studies that observed Canton-S flies in tiny mating arenas suggested that ovipositor extrusions may include both rejection and acceptance signals^44,45^. Further research is needed to elucidate the gustatory and olfactory components associated with ovipositor extrusion and the information they convey to males about females’ likelihood of mating. It is, however, possible that highly motivated males elected to ignore females’ ovipositor extrusion even when it unambiguously signalled rejection.

Females sometimes responded to a pursuing male by walking or running on the fruit. I did not record walking or running because, based on laboratory studies, they are considered integral components of female behavior during courtship^46–48^. In the natural settings, however, it appeared that the commotion associated with a female running away from a pursuing male led to other males occupying the fruit to join the pursuit, resulting in a few males simultaneously and excitedly courting a single female (Supplementary Video S1 4:00). Paradoxically then, the apparent female evasion led to further pursuit by more males. Theoretically, such simultaneous pursuit could enhance females’ abilities to choose males based on both male–male interactions, which could lead to the best males dominating courtship^49^, and the females’ abilities to directly compare males. Importantly, these field observations suggest that laboratory protocols involving one female and multiple males are more realistic than protocols including a single female and a single male.

### Male–male courtship

Male–male courtship has been well documented in laboratory studies of fruit flies primarily in the context of mature males pursuing young males, who still do not possess the complement of cuticular hydrocarbons that characterizes sexually mature males^50–53^. The field setting, however, was rather different as all males were mature and experienced in interacting with other mature males and females. Nevertheless, I observed many bouts of male–male courtship (Supplementary Video S1 5:05). It seems that males in the field often adopt the strategy of approaching a fly with courtship before recognizing the sex of that fly. Sometimes, they are rather slow to recognize the sex of the fly they court.

### Mating frequency

I recorded a low mating rate of 0.19 matings per hour in a natural setting where males performed 29 ± 3.2 courtship bouts per h. These values indicate a very high ratio of sexually receptive males to females, which is consistent with previous reports from field studies. Most notably, two studies reported no or very rare cases of courtship leading to mating in natural settings^33,34^. It is fair to assume that all females in my study were non-virgin as I allowed newly eclosed flies to acclimate for a few days before commencing my observations. My video recordings, which coincided with peak mating activity, covered half the grapes. I thus assume that I captured about one quarter of the daily matings on video (because I recorded at half the available space and half the available time). This translates into a mating rate of about 0.6 per female per day (based on presumed 44 matings per 25 females over 3 days). This value is similar to the most reliable values taken from natural settings, which suggest that females remate every few days^33,54,55^. While this estimate is much lower than the values from laboratory studies, it is now well established that non-overlapping generations, and the confined space with no refuge typical to most laboratory experiments, lead to overestimation of the natural levels of mating rates^56–58^.

### Mate choice copying?

I saw no evidence that females approached to observe copulating flies, while males frequently inspected mating couples in their relentless search for mating opportunities. These observations are just what one would expect in the natural settings of a highly male-biased operational sex ratio. Given that females are persistently pursued by many males from the moment they eclose^59,60^, they can have excellent information about the features of males in their surroundings, which they can rely on for choosing their first and subsequent mates^61,62^. Furthermore, because matings in the field are infrequent and scattered in space, females seeking copulating flies would need to expend significant time and energy to locate them. Hence the females should focus on searching for the best sites for feeding and egg laying given that the information about prospective mates reaches them at an exceedingly high rate. As for the males under the setting of a highly male-biased operational sex ratio, they should simply mate with any willing female. It is difficult to imagine realistic natural scenarios where male fruit flies would benefit from rejecting a female willing to mate. It follows that, if males cannot afford the luxury of choosing mates, other males have no opportunity for mate choice copying. In short, the current data suggest negligible role for mate choice copying in natural settings although it may occur in situations not captured in my setup.

### Temperature

Perhaps the most striking of my observations was flies’ resilience to extreme temperatures. On the cold side, I initiated the compost and its associated *D. melanogaster* population in January, when the average low and high temperatures were 4 °C and 19.7 °C respectively, and collected by aspiration active flies adept at evading me at temperatures below 10 °C. I conducted the first 2 replicates of the field work in April, when the average low temperature was 12.3 °C. Moreover, I recorded peak fly activity early in the morning, when temperatures were close to the daily lows (Fig. 2). Partridge et al.^34^ also reported peak fly activity in the field at dawn in May in Davis, CA, where the average low temperature in May is 12.7 °C. It thus appears that flies prefer early morning activity in spite of the relatively cold temperatures. The early morning activity does not seem to be an outcome of some circadian rhythm constraint because fruit flies in Arizona do switch to an afternoon peak of activity in the winter^31^. Peak fly eclosion is at dawn^63^ and females reach sexual maturity within about 24 h^64,65^. Hence the peak availability of virgin females reaching sexual maturity may be in the early morning. This can readily explain the morning peak of male activity. I observed no fly activity at night even in May, when the average low temperature was 18.2 °C, so it is possible that the standing crops of yeast peak early in the morning. This may explain females’ preference for early morning feeding. It is also possible that diurnal variation in predation risk favors early morning activity, but this will require future quantification in the field.

The sizable, primarily lab based, literature on fruit flies’ responses to temperatures indicates a strong preference for temperatures around 25 °C^66–69^. Much of the relevant research has focused on survival under either extreme cold or acute heat. Generally, the lab-based experimental data suggest that flies do not handle well temperatures above 30 °C and below 0 °C. There is, however, significant latitudinal variation, with flies from colder climates surviving freezing longer and flies from warmer climates surviving extreme heat longer. Furthermore, acclimation enhances survival at extreme temperatures, and high humidity enhances survival at high temperature. Nevertheless, most flies die rapidly at temperatures above 35 °C and at or below 0 °C^70–76^. We know, however, too little about flies’ handling of temperature under natural settings in different climates.

I suggest that flies in natural settings are more adept at handling extreme temperatures than laboratory studies have led us to believe. It appears that flies have a strong preference for early morning activity, most likely owing to the associated fitness benefits discussed above. Hence flies will show peak morning activity even at temperatures in the low teens. Flies’ preference for early morning activity is well suited for warm climates, as temperatures are at their minimum at this time. Based on the laboratory studies mentioned above, I was worried about the flies managing the high heat in May, when mid-morning temperatures quickly climbed above 30 °C. The flies, however, appeared to manage well temperatures between 35 and 40 °C. While flies sometimes remained immobile on the wet towels for much of the day (Fig. 1c), they most often went into hiding. Although I searched for these flies, I avoided disturbing them and thus have no quantitative data about their distribution. The two hiding sites I identified, however, were the undersides of the stools and the insides of the narrow tunnels within the cardboards at the rear of the stools (Fig. 1a,b). Both these sites are well shaded and protected from air movement, which can help reduce water loss in the dry air. They likely also provide good protection from predators. These spots probably reflect well on places that flies innately seek at high temperatures when away from human habitation. The behavioral thermoregulation I observed has been well studied in ectotherms, primarily reptiles^77,78^. Given their role as a leading model system, fruit flies could serve as an excellent model system for research on behavioral thermoregulation in natural settings and its fitness consequences, especially in the pertinent context of global warming.

In conclusion, I documented for the first time the occurrence in the field of both significant sociability and fair levels of male–male aggression in fruit flies. Further research will be necessary for quantifying the dynamics that underly these social behaviors in natural settings. Similarly, in spite of significant, primarily laboratory based research, we do not fully understand the subtleties of interactions between males and females at fruit. Specifically, we do not know the factors that determine males’ allocation of pursuit effort to different, previously mated females, and females’ differential abilities to deflect male advances. Finally, the factors that cause fruit flies to concentrate activity in early mornings even at cold temperatures as well as their ability to sustain extreme heat require future quantification.

## Methods

### General

I conducted the study at the yard of my temporary residence in Tucson, AZ, USA in spring 2020. In January, I established a compost supplied frequently with my family’s organic kitchen scrap. Fruit flies (*Drosophila spp*) promptly occupied the compost and numbered several hundreds when I collected females in early March. I wished to conduct the research only on a single species. *D. melanogaster* and *D. simulans* females, however, look identical^79,80^. I thus had to establish a pure *D. melanogaster* population in the laboratory and release early descendants from that population back into a confined area in the field. Early on a few cold mornings (~ 8 °C), I aspirated females from the compost and brought them to the laboratory. I anesthetized the females with CO_2_, inspected them under a microscope, and placed each *D. melanogaster / D. simulans* female individually inside a standard vial containing 10 ml fly medium and a sprinkle of live yeast. After offspring eclosion, I inspected males’ genitals and discarded all vials containing *D. simulans*. I placed offspring from about 50 *D. melanogaster* vials inside a population cage with screen walls 30 cm wide (BugDorm 1, BioQuip), and supplied 2–4 fresh bottles each containing 50 ml fly medium and a dash of live yeast every few days. Newly eclosed flies from these bottles served as subjects in the field observations.

For a few reasons, I had to conduct the field work within an enclosed area. First, as noted above, this was the only way that I could exclude the identically looking *D. simulans* flies. Second, it was essential that I also exclude other fruit fly species to avoid distracting inter-specific interactions, which could include displacement of *D. melanogaster* from fruit. Third, I wished to exclude other insects, spiders, lizards, birds and mammals, which were abundant in the area. Finally, I wanted to control the fly density in order to ensure a manageable setting for quantifying behavior. Hence I conducted all observations inside a tent measuring 3 m by 3.6 m and 2.15 m high (Core Instant Screen House Canopy). The walls of the tent were made of screen while the roof was an opaque, waterproof fabric. Along the south side of the tent, I placed four stools 60 cm high and 40 cm wide. I randomly assigned stool location in each replicate. The feet of the stools were inside plastic containers filled with water to exclude crawling animals, primarily ants. I covered the tops of the stools with towels and attached to their rear sides vertical 40 cm by 40 cm cardboards, which provided shelter. To add further complexity and refuge, I glued several pipe cleaners to each stool top and cardboard (Fig. 1a,b). In the May replicates, I soaked the towels on the stools with water to compensate for the low humidity. Finally, I placed a thermometer under one of the stools, obtained daily maximum temperatures from the weather station at the University of Arizona located about 3 km from the study site, and monthly average low and high temperatures from the National Oceanic and Atmospheric Administration.

From mid-April to late May, I conducted five replicates. On day 1 of each but the first replicate, I released 25 newly eclosed flies of each sex. In replicate 1, I released 40 newly eclosed flies of each sex. Prior to fly release, I placed two store-bought small grapes (*Vitis vinifera*) at the rear of each stool and 20 cm apart. The grapes were purple in the two April replicates and green in the three May replicates. I switched from purple to green grapes in order achieve a higher contrast between the flies and their background. I chopped the top of each grape to expose flesh and added a drop of live yeast and orange juice suspension. I kept adding a drop of the suspension to each grape at dawn on each morning of the study. At the right side of each stool, I placed an iPod mounted to a tripod for video recording of the right grape (Fig. 1b). I allowed flies to mature and acclimate to the enclosure settings and initiated observations when they were 3–5 days old.

Each replicate comprised three days of live observations on flies at the eight grapes, and video recordings of four grapes. I conducted observations from dawn to dusk but focused on the early mornings, which were the only periods of significant fly activity. On 5 days spread over 3 replicates, I conducted scans from dawn to dusk. On all 15 days of all replicates, I performed scans during 3 early morning time periods that corresponded to peak fly activity. Each scan involved gently approaching to within about 20 cm of each grape and counting all individual flies of each sex. In addition, I recorded all flies seen away from the fruits. I noted all activities including courtship, aggression and mating. To accommodate the large increase in temperature between the April and May replicates (Fig. 2a), the 3 early morning scans were at 7:30, 8:30 and 9:30 in the two April replicates, and at 6:30, 7:30 and 8:30 in the three May replicates. Similarly, I conducted the video recordings between 7:30 and 8:30 in April and between 6:30 and 7:30 in May. Females laid many eggs on grapes, which I reared to adulthood during 2 replicates, but I did not record egg laying activity. At the end of each replicate, I placed traps in the enclosure, removed all flies and froze them.

### Fly numbers

During the scans of all the grapes, I counted all fly numbers primarily in order to calculate the sociability index described in the next section. Fly numbers at fruits, however, are also relevant for our understanding of the daily dynamics of fly activity and social behavior in natural settings dominated by large daily and seasonal variations in temperatures. I thus graphed the results of the whole day scans (n = 5 days) and analyzed fly numbers by time period and sex for the 3 early morning scans (n = 15 days). I used a square root transformation of the fly numbers to achieve normality and analyzed the data set with a general linear mixed model (GLMM) with replicate as a random factor, sex, time period and day as fixed factors and all two-way interactions.

### Sociability

Here I tested the prediction that flies would prefer to be in groups than alone. To test this prediction, I conducted 3 hourly counts of the number of flies at each of the 8 grapes as detailed above. From these numbers, I calculated the sociability index, which my students and I have successfully used to quantify sociability in both larval and adult fruit flies under controlled laboratory conditions^26,81^. The index is a standard measure^82^, which is calculated as the variance in the number of flies at each grape divided by the mean number of flies at each grape. A value of 1 (mean = variance) represents random distribution, a value of 0 implies uniform distribution, and any value significantly above 1 indicates sociability. A larger sociability index means greater tendency of flies to form groups. See illustrations in Durisko et al.^26^ and Scott et al.^81^. I log transformed the sociability data to achieve normality and analyzed them with a GLMM with replicate as a random factor, time period and day as fixed factors and all two-way interactions. Overall, the sociability scores were based on 5 replicates each lasting 3 days each. As a follow up aimed to glean insights into the dynamics of sociability, I watched again all 12 videos of the last replicate and recorded all arrivals and departures of flies from each grape.

### Fly behavior

Every morning, I conducted 1-h video recordings of 4 out of the 8 grapes. I lost 1 video owing to error so the data set included 59 h of fly behavior, which I scored using BORIS^83^. The video scoring involved recording of all interactions between flies.

### Aggression

My major goal was to assess whether aggression is an important factor shaping fruit fly social interactions in natural settings. Much of the aggression was between males and consisted primarily of lunging, characterized by the aggressor making a fast movement and hitting with his head his opponent, the receiver of aggression. I also recorded the occasional events of boxing, where both flies stand up on their hind legs and strike their opponent with their forelegs (Supplementary Video S1 0:51). Wing threat, in which the aggressor raises his wings at 45° towards his opponent^5^ was infrequent and is not reported here. After each aggressive encounter, I noted the receiver’s response. On the two extremes, the receiver could either fly away or respond with aggression. Intermediate responses included retreating from the aggressor by walking or running but remaining relatively close on the small grape; staying put, effectively ignoring the aggressor; and wing waving, which is males’ typical response to males that court them^84^. To assess variation in lunging frequency over days, I constructed a GLMM with replicate as a random factor and day as a fixed factor. Because boxing was rare, I conducted only descriptive statistics on it. Similarly, I conducted only descriptive statistics on the males’ response to male aggressors.

My students and I previously reported about the occurrence of subtle aggression among males, which involved courtship interference. The interfering male positions himself between a female and the male pursuing her and takes over courtship^49^. While a systematic study of courtship takeover requires controlled laboratory settings, I wished to assess whether such behavior occurs in natural settings. Hence I recorded all cases of male–male interactions during courtship as well as mating.

In addition to the prevalent male–male aggression, I also recorded rare cases of female-female aggression. Finally, because I scored all fly interactions, I noticed novel instances of aggression between males and females, which I report as well.

### Sexual behavior

Because almost all the data on fruit flies’ sexual behavior are from the laboratory, I wished to quantify activities related to reproduction in the field. I recorded all bouts of males courting females at fruit. Courtship included all following, singing, and mounting attempts^85,86^. To assess changes in courtship over time, I calculated the proportion of time that males at each grape spent courting females, used an arcsine square root transformation, and constructed a GLMM with replicate as a random factor and day as a fixed factor. I also noted obvious females’ responses to the pursuing males, which included wing waving, ovipositor extrusion, and flying away. Wing waving consisted of vibrating the wings, which, most likely, produced a rejection signal^84^. The ovipositor extrusion involved either the ovipositor or an egg protruding from the tip of the abdomen^42,43,87^. Females often ignored the males and continued feeding, or either walked or ran, but I did not record these behaviors. An unexpected observation was the prevalence of male–male courtship. To estimate the relative prevalence of this behaviour, I counted all male–male courtship bouts in 1 h and compared their frequency to that of male–female courtship bouts. Finally, laboratory studies reported that fruit flies copy the mate choices of others^88–90^. To assess the relevance of that behaviour in the field, I quantified all approaches to and interactions of other flies with all copulating flies for the whole durations of their matings. I defined approach as anything closer than a head width—about 1 mm. Approaches and interactions could indicate the potential for focal flies to acquire information about the copulating flies, which they could subsequently use for mate choice.

## Supplementary Information

Supplementary Video.

Supplementary Information.
