# Supplementary material for: Natural history of social and sexual behavior in fruit flies
Source: Sci Rep. 2020 Dec 14;10:21932. doi: 10.1038/s41598-020-79075-7 (PMC7736333; doi:10.1038/s41598-020-79075-7)
Supplement: Supplementary file 2 — Supplementary Information. [file 41598_2020_79075_MOESM2_ESM.pdf]

Natural history of social and sexual behavior in fruit flies

Reuven Dukas

Animal Behaviour Group, Department of Psychology, Neuroscience & Behaviour, McMaster University, 1280 Main Street West, Hamilton, Ontario, L8S 4K1, Canada

Email: dukas@mcmaster.ca

Caption for the Video. Selected clips that illustrate key behaviours of fruit flies in natural settings. (a) Sociability. On grape B, note the males' continuous patrolling in pursuit of females, and females' ovipositor extrusions. (b) Aggression. In clip 1, note that the receivers of aggression stay on the fruit while in clip 2, all 3 receivers of aggression depart the fruit. Clip 3 shows a subtle courtship takeover while clip 4 depicts courtship takeover that leads to mating. Note that the losing male continues courting after the takeover. Clip 4 shows female-female aggression, which is rare and subtle compared to male-male aggression. (c) Sexual behavior. The simultaneous pursuit of females by multiple males was common. In clip 2, note the successful evasive behaviors by the female. In clip 3, male-male courtship was rather long. Male-male courtship events were frequent but typically brief.
